# Psychological impacts of maternal migration on left-behind children: a cross-cultural review

**DOI:** 10.3389/fpsyg.2024.1407733

**Published:** 2025-01-15

**Authors:** Dana Al-Azzeh, Jasmin Lilian Diab

**Affiliations:** ^1^Institute for Migration Studies, School of Arts and Sciences, Lebanese American University, Beirut, Lebanon; ^2^Department of Communication, Mobility and Identity, School of Arts and Sciences, Lebanese American University, Beirut, Lebanon

**Keywords:** attachment disorder, migrant workers, mental health, coping, strategies, trauma, cultural practices

## Abstract

In the pursuit of a brighter future for their children, many female migrant workers leave their home countries to seek employment abroad, often as caregivers for families in wealthier nations. This decision necessitates prolonged separation from their own children, depriving them of the opportunity to witness and participate in crucial stages of their children’s development. The absence of these mothers, combined with inconsistent caregiving in their stead, significantly increases the vulnerability of left-behind children to mental health challenges, such as depression, anxiety, and difficulties in forming emotional bonds with others. Moreover, even after the migrant mother’s eventual return, the disruptions in attachment often result in long-term consequences, including strained and distant relationships with their children. This article explores the complex impact of maternal migration on the mental well-being of left-behind children across various cultural contexts. It highlights key findings, such as the role of communal caregiving practices in mitigating adverse effects in some societies, while noting that these practices are not a complete solution. The study underscores the need for culturally sensitive mental health interventions and policy measures to support the well-being of children affected by maternal migration globally.

## 1 Introduction

The feminization of migration has not only increased the visibility of women as economic contributors but has also brought to light the unique challenges they face as both workers and caregivers (Piper, 2005). As women increasingly migrate to support their families, often taking on roles as primary breadwinners, the traditional family structure undergoes significant changes (Thao and Agergaard, 2012). This shift can lead to a reconfiguration of gender roles within the household and impact the caregiving dynamics, with significant implications for the emotional and psychological development of the children left behind (Lam et al., 2013). Understanding the feminization of migration is thus crucial to comprehensively addressing the social and familial consequences of maternal migration in diverse cultural contexts.

Mothers play many different roles in the lives of their children, including teacher, playmate, disciplinarian, caregiver and attachment figure. Of all these roles, their role as an attachment figure is one of the most important in predicting the child’s later social and emotional outcome (Benoit, 2004; McLeod, 2009). In recent years, participation of women in international migration has increased (Castles, 2017). This phenomenon of “feminization of migration” has altered traditional gender roles and dynamics both within migrant families and in the labor markets of both sending and receiving countries (Piper, 2013). The migration of a mother, often in search of better economic opportunities, can disrupt the attachment relationship between the mother and her child, leading to lasting emotional and psychological effects (Bălțătescu et al., 2023; Brumariu et al., 2020; Adem, 2021; Ee, 2011; Cappelloni, 2011). The study of attachment of children with their mothers was established by “white upper class men” who were separated from their mothers to join boarding schools (Van der Kolk, 2014, p. 129). According to attachment theory, it is highly possible for children who have had their contact cut off with their mothers to develop unstable attachment and consequently have poorer mental health than those who had stable contact with their mothers (Yang, 2022). It is worth pointing out that transitional families originate typically from the Global South where different parenting styles prevail. This includes the presence of multiple caregiving relationships (Mazzucato et al., 2015). For this reason, it can be argued that not all children in different cultural contexts will have the same emotional reaction when separated from their mothers. Hence, here we review empirical evidence from different cultural contexts to explore the effect of mothers’ migration on the psychological wellbeing of children, and the factors that exacerbate or mitigate the absence of the migrant mother on her children in different cultural contexts. Articles were selected from the PsychINFO database and Google Scholar. Google Scholar served as a valuable starting point for identifying relevant literature on the topic. We then utilized PsycINFO, an international database renowned for its comprehensive and current resources in psychology and social sciences research. To refine our search, we employed keywords such as “children of migrants”, “left-behind children”, “attachment”, “infant separation”, and “transitional families.” Advanced search techniques, including Boolean logic, wildcards, and truncation, were applied to optimize our results in PsycINFO. To achieve the objectives of this mini-review, the following criteria for selecting publications for the analysis were formulated: (see Appendix 1 for the explicit search strategy).

1. The articles are of empirical nature. Thus, review articles were excluded. Empirical studies provide reliable and valid information. Depending on empirical studies benefit the review in so many ways among which is making it more reliable and valid because data collected through empirical research is less biased, and it is difficult to verify the accuracy of data in non-empirical research, review articles were excluded as we must revisit the main articles included in the review, as accurately assessing their quality is impossible without thorough review.
2. Only studies that dealt with international migration were included, studies that dealt with internal migration were excluded because migrants who migrate internally have the chance to conduct regular visits to their children, unlike international migrants who only visit there every two years depending on the contracts they have. This difference may yield different outcomes for children.
3. We included studies that compared children who have mothers as migrants, children who have fathers are migrants, children who have both parents as migrants, and children who live with both parents to allow accurate comparison.
4. We included research articles conducted in different countries and different cultural contexts to allow cross country comparison.

This review addresses several critical gaps in the existing literature on the impact of maternal migration on children’s psychological well-being. Firstly, much of the previous research has focused predominantly on Western contexts, often neglecting the diverse cultural backgrounds from which many migrant families originate. By including studies conducted in different countries and cultural contexts, this review seeks to fill the gap in understanding how the effects of maternal migration may vary across different cultural settings, where parenting styles and family structures differ significantly. Secondly, traditional attachment theory, which underpins much of the existing research, has primarily been developed based on experiences from Western, upper-class contexts. This review challenges the universality of these findings by exploring how attachment and emotional outcomes may differ in non-Western contexts, particularly in families from the Global South where multiple caregiving relationships are more common.

Moreover, this review addresses the lack of comparative analyses between different family configurations—such as children with migrant mothers, migrant fathers, both parents as migrants, and those living with both parents. By including studies that offer these comparisons, the review provides a more nuanced understanding of how different parental migration scenarios uniquely impact children’s well-being. Finally, the review also responds to the scarcity of empirical studies that specifically examine the long-term psychological effects of maternal migration on children, beyond immediate or short-term outcomes. By focusing on empirical research, this review contributes to a deeper understanding of the lasting emotional and psychological implications of disrupted maternal attachment due to migration.

## 2 Mental health implications: the significance of maternal influence

The effect of parental migration on Left-Behind Children (LBC) has been an issue of debate among scholars from different disciplines, namely public health, psychology, anthropology, migration studies, among others. While some studies concluded that parents’ decision to leave and work overseas comes at a heavy emotional and psychological cost to the left behind children (Bragin and Pierrepointe, 2004), others concluded that the psychological consequences of parents’ migration are not always unfavorable (Asis, 2006; Vanore et al., 2015). According to both schools of thought, the outcome of parental migration depends on different factors among which is the gender of the migrant parent (Antia et al., 2020). Evidence was found in the literature that the migration of mothers has a higher cost on children than fathers’ migration. According to Adhikari et al. (2014) it was found that the migration of Thai mothers has a significant impact on the mental health of their left-behind children in Thailand. According to the study, children of migrant mothers feel “lonely, angry, unloved, afraid and anxious in comparison with children of non-migrants, or children of migrant fathers.” The aforementioned findings corroborate with Graham et al. (2012) findings, the study was conducted on LBC living in the Philippines and Indonesia, the study revealed that Indonesian children whose mothers migrated were more likely to experience “happiness deficit” when compared with those living with both parents or those who have their father as the migrant and stay in the care of their mothers. Similarly, in their cross country study conducted in Nigeria, Angola, and Ghana, Mazzucato et al. (2015) reported that children’s psychological wellbeing is negatively affected when mothers migrate and children live with their fathers in Nigeria and Angola, but this was not the case for LBC in Ghana. A study conducted in Zimbabwe reached a similar conclusion, the study reported that children cope better when their fathers migrate, as remaining mothers provide their children with adequate care. Furthermore, children in this study do not perceive paternal migration as a negative thing if their mother is around to care for them (Manyeruke et al., 2021). The work of Cardoso et al. (2021), Ukwatta (2010), Sapkota (2020), Hugo and Ukwatta (2010), and Battistella and Conaco, 1998 support the findings of the aforementioned studies.

## 3 Identifying factors: why maternal migration may have a greater impact

Various studies conducted in different countries and cultural contexts have consistently found that children left behind by migrant mothers experience more mental health issues compared to their peers. This raises the question of whether the absence of mothers has a similarly detrimental effect on left-behind children across cultures. Factors such as cultural norms, parental roles, quality of substitute care, stability of arrangements, level of contact with the migrant parent, and the child’s age all play significant roles in determining the impact of maternal migration on these children (Hugo and Ukwatta, 2010; Wanninayake, 2016; Battistella and Conaco, 1998). The studies suggest that in certain cultures, maternal migration is seen as more harmful than paternal migration. Key reasons will be discussed below.

### 3.1 Stability in caregiving arrangements for migrant mothers’ children

In their cross-country study conducted in Ghana, Nigeria, and Angola, Mazzucato et al. (2015) concluded that migrant mothers often struggle to find stable caregiving arrangements for their left-behind children, resulting in the children frequently changing caregivers. This constant shifting of caregivers contrasts sharply with situations where the father migrates, as caregiving arrangements tend to be more stable when mothers remain in the household. The instability in caregiving has profound negative outcomes for left-behind children, as it hampers their ability to form stable attachment bonds. Stable and secure attachment bonds with caregivers are crucial protective factors against the deterioration of psychological well-being (Van der Kolk, 2014, p. 131). Children who are repeatedly exposed to different caregivers may experience heightened levels of anxiety, insecurity, and confusion, as they struggle to establish a consistent sense of trust and emotional security. These children may develop attachment disorders, exhibit behavioral problems, and face difficulties in forming healthy relationships later in life.

Manyeruke et al. (2021) reached a similar conclusion in their study on children in Zimbabwe, noting that children cope better psychologically when their fathers migrate rather than their mothers. In fact, the study found that these children often have better psychological well-being than those living with both parents. The authors attribute this to the mothers’ ability to maintain family cohesion and provide consistent emotional support, which is critical for the child’s development. In many cultures, mothers are seen as the emotional anchors of the family, and their presence provides a sense of stability and security for children. When the mother is the caregiver, the likelihood of consistent caregiving practices increases, which helps children adapt more effectively to the absence of the father.

In a similar vein, a study by Graham et al. (2012) explored the experiences of left-behind children (LBC) in Indonesia and the Philippines and found that children of migrant mothers in Indonesia are less likely to seek emotional support from adults when they encounter difficult problems. This reluctance to seek support is closely tied to the disruption of support networks that occurs when mothers migrate. In many Southeast Asian cultures, the mother is often the central figure in a child’s emotional support system. Her absence can lead to a significant weakening of these support networks, leaving children without a reliable adult to turn to during times of distress. The absence of the mother, who is typically more attuned to the emotional needs of the child, creates a gap that is not easily filled by other family members or caregivers, particularly in societies where the extended family may not be as involved in day-to-day caregiving.

Different cultures handle caregiving in various ways, and these practices have significant implications for children’s psychological well-being. In African cultures, for example, communal child-rearing is a common practice, where caregiving responsibilities are shared among extended family members or the wider community (Mazzucato et al., 2015; Manyeruke et al., 2021). While this approach can provide a safety net for children when their mothers migrate, it also introduces the risk of instability if caregiving arrangements frequently change. In contrast, in Southeast Asian cultures, caregiving is often more centered on the nuclear family, with the mother playing a pivotal role in the emotional and psychological development of the child (Reyes, 2007) The migration of the mother in such contexts can therefore have more pronounced negative effects, as the child loses their primary source of emotional support.

Moreover, the frequency of caregiver changes can lead to a sense of instability and unpredictability in the child’s life, which is detrimental to their mental health (Freud and Burlingham, 1943; Bowlby, 1951, 1973) Children who are unable to form secure attachments with a consistent caregiver are at greater risk of developing emotional and behavioral issues, such as anxiety, depression, and difficulties with social interactions (Makariev and Shaver, 2010). The lack of a stable caregiver can also impact a child’s ability to develop trust in others, which is fundamental to their overall psychological resilience (Harden, 2004).

### 3.2 Cultural norms: examining the African context

In many African countries, child-rearing is not solely the responsibility of biological parents but is instead a collective effort that involves extended family members and the broader community. The well-known African proverb “It takes a village to raise a child” epitomizes this deeply ingrained practice, where community members collectively teach children societal norms, values, and traditions (Serra, 2009). In this context, child fostering, where children are raised by non-biological parents—often relatives or close community members—is a common and accepted practice. This communal approach to child-rearing can significantly influence the outcomes of parental migration on African children, as being raised by a non-biological parent is not seen as unusual or stigmatizing (Poeze and Mazzucato, 2013).

For example, in West African countries like Ghana and Nigeria, children who are left behind by migrating parents often continue to live with relatives, such as grandparents, aunts, or uncles. This form of social parenthood, where caregiving responsibilities are distributed among multiple adults, is prevalent across the region and serves to buffer the potential negative impacts of parental absence. Mazzucato et al. (2015) found that in these contexts, children do not experience stigma due to their parents’ migration and the subsequent care they receive from extended family members. The norm of communal caregiving means that these children are still embedded within a supportive community structure that provides continuity and emotional security, despite the physical absence of their parents.

However, this positive outlook on communal child fostering is not without its challenges. In the same study, Mazzucato et al. (2015) noted that the frequent changes in caregivers, which can occur when children are passed between different relatives, can undermine the stability necessary for secure attachment. In Nigeria and Ghana, for instance, while communal child fostering is generally seen as beneficial, the lack of consistent caregiving due to frequent caregiver changes resulted in negative psychological outcomes for children. These outcomes included feelings of insecurity and difficulties in forming stable attachment bonds, highlighting that even within a communal caregiving context, the stability of the caregiving arrangement is crucial for the child’s mental and emotional well-being.

This raises important questions about whether more stable caregiving arrangements could enhance the positive aspects of communal child fostering and better mitigate the negative effects of maternal absence. Further research is needed to explore this potential and to determine how stable, consistent care can be maintained in contexts where communal child-rearing is the norm.

The need to consider the historical and social context is further underscored by findings from Angola, where Mazzucato et al. (2015) discovered that left-behind children exhibited poor mental health and well-being outcomes regardless of whether the mother or father migrated. This phenomenon is closely tied to the traumatic legacy of the Angolan Civil War, which devastated many communities and weakened the social fabric that typically supports communal child-rearing. The war-induced trauma significantly diminished social solidarity, leading to less effective care for left-behind children. In this context, the communal child-rearing practices that might have otherwise provided stability and support were severely disrupted, demonstrating how historical events can profoundly influence the effectiveness of cultural practices like communal caregiving.

Comparatively, in Zimbabwe, Manyeruke et al. (2021) found that children in both transitional (with migrant parents) and traditional families (with both parents present) showed no significant differences in attachment styles, with secure attachment being the most common. This finding was attributed to the societal norms of communal child fostering, which are deeply embedded in Zimbabwean culture. The study suggests that, unlike in Angola, the societal structures supporting communal caregiving in Zimbabwe have remained intact, allowing children to maintain secure attachments even in the absence of their biological parents. However, the study also noted that children tended to cope better with paternal migration than with maternal absence, indicating that the role of the mother as a primary caregiver is still crucial, even within a communal context. This observation calls for further research to clarify whether secure attachment is more strongly associated with children of migrating fathers or mothers and to better understand the nuances of attachment formation in communal caregiving settings.

When comparing these cultural norms to other regions, such as Asia or Latin America, distinct differences emerge. In many Southeast Asian cultures, like those in Indonesia and the Philippines, caregiving tends to be more centered on the nuclear family, with the mother often playing a pivotal role in the emotional and psychological development of the child (Yeoh et al., 2020) The migration of the mother in these contexts can therefore have more pronounced negative effects, as the child loses their primary source of emotional support, and extended family members may not be as involved in caregiving. In contrast, in Latin American cultures, extended family networks also play a significant role, but the emphasis on the nuclear family remains strong, and the mother’s role is similarly central (Martinez and Acosta Gonzalez, 2022). The migration of mothers in these contexts often leads to challenges in maintaining consistent and supportive care, similar to the challenges observed in African settings, albeit within a different cultural framework (Pharr et al., 2014).

### 3.3 The quality of care provided by caregivers

Evidence was found in the literature that the quality of care LBCs receive from their substitute caregivers can lead to a paradigm shift in parental migration outcomes. Substitute caregivers can be both protective factors and risk factors for LBCs depending on the quality of attachment bond they form with LBCs (Battistella and Conaco, 1998). Attachment bonds are founded on the quality of care, love, attunement, and nurture the children receive from their caregivers (Van der Kolk, 2014, p. 132). The effectiveness of substitute caregivers can vary widely, as they may not always be able to replicate the nurturing and care typically provided by the mother. This can lead to gaps in care and support for children which will consequently affect their mental health. On the other hand, some substitute caregivers succeed in fostering a secure attachment with LBCs by being loving, nurturing, and caring; this can mitigate the psychological consequences of mothers’ absence on children, and help these children lead healthy and happy lives (Hugo and Ukwatta, 2010; Lam and Yeoh, 2019). Adhikari et al. (2014) pointed to the strong association between the age and the mental health status of the substitute caregivers with the psychological wellbeing of LBC in Thailand. In this study, it was found that the mental health of substitute caregivers significantly impacts the psychological well-being of left-behind children (LBC). LBCs under mentally healthy caregivers exhibited better mental health compared to those under mentally unhealthy ones, who may be incapable of providing suitable care and could create stressful or abusive environments. Additionally, younger caregivers below thirty often prioritize personal lives and careers, leading to neglect of LBCs. In this regard, it is also notable that Graham et al. (2012) indicated that children who were left in the care of older grandmothers do not get the appropriate emotional support they need when they face problems, because older grandmothers tend to lack the necessary knowledge about the emotional needs of these children who belong to a much younger generation, which can affect their psychological well being. In summary, the studies emphasize that the age appropriateness of caregivers can immensely affect the mental health outcomes of LBCs.

The quality of care received by left-behind children affects reunions with their mothers. Those with secure attachments to substitute caregivers may find the return of their mothers stressful due to separation from familiar caregivers and routine disruption. Conversely, children with neglectful or abusive caregivers may struggle to trust their mothers, making reunions and living together difficult (Bragin and Pierrepointe, 2004).

### 3.4 The age of the child

The impact of parental migration on left behind children can vary significantly depending on the age of the children at the time of separation. Adhikari et al. (2014) concluded in their study that younger children in Thailand feel abandoned by their mothers when they migrate, but older children accept their mothers migration because they start to fully grasp the reasons behind it. Bragin and Pierrepointe (2004) partially disagree with Adhikari et al. (2014)’s findings. The authors argued that older children do not readily accept their mothers’ migration without mental impact. They struggle to understand why their mothers leave for better opportunities abroad, leading to increased distress as they prioritize love and care over material possessions.

Studies in the African context have yielded different findings. Cebotari et al. (2017) reported that older children in African transnational families find themselves more vulnerable in terms of health conditions than younger children. The same study found that age is a statistically significant indicator in relation to health – older children tended to rate their health more negatively in both Ghana and Nigeria. On the other hand, Adhikari et al. (2014) found the opposite in her study that was conducted in Thailand. The study concluded that older children enjoy better mental health outcomes than younger children who are in transitional households, because they become less dependent on their mothers as they expand their support network as they grow. Noteworthy, Nguyen (2016) emphasized the importance of child development between the ages of 5 and 8 years old, and argued that leaving children behind at this crucial age delays cognitive development. This was the case for Vietnam, India, and Peru, but not for Ethiopia, as the study concluded that parental migration does not have a significant effect on children there. This can be attributed to the common practice of communal child fostering in Africa.

### 3.5 Perceived gender roles in child rearing

In many cultures, mothers are considered the primary caregivers for their children, and have the major responsibility in child rearing and household management (Bornstein and Putnick, 2016; Zimmermann et al., 2022; Leemann et al., 2020; Sharma et al., 2016). The work of Graham et al. (2012) showed the perception of LBCs regarding this issue. Children who were interviewed in this study, had gendered understanding of caring norms, one of the children who was interviewed for the aforementioned study stated the following about her father:

*“[…] He’s the one who cooks breakfast for us, and when I’m sick he’s the one who takes care of me. He’s the one who launders the clothes. He’s okay but it’s different if the mother is the one caring. Because the mother is of course the light of the home. Because Papa is doing a woman’s work. He should be doing manly work right? Mama is the one doing the man’s work.”*

LBC evaluation of their well-being can be affected by the gender-normative discourses. They experience mother care, father care, and care from other female caregivers differently (Lam and Yeoh, 2019). Furthermore, mothers’ migration disturbs these gendered norms which lead to social stigma or judgment, both for the mother and the children, potentially leading to feelings of guilt, shame, or social isolation (Parreñas, 2005, p. 5).

### 3.6 Maintaining contact with the mother: is it a protective factor?

Evidenced in the literature is the reality that the emotional well-being of children of migrant mothers may improve if they keep in contact with their migrant mothers. Studies also found that feelings of intimacy and attachment are likely to be disrupted when contact with a migrant parent is rare (Graham et al., 2012; Manyeruke et al., 2021). New technologies have made communication between LBCs and their migrant mothers easier. Many migrant mothers contact their children frequently and provide them with the emotional support they need from a distance (Boccagni, 2012). However, the place of origin of the migrant worker plays a key role in maintaining communication with their children. These new means of communication are not always available in the migrant worker’s place of origin. For example, Ukwatta (2010) concludes that Filipino migrant workers make more use of new technologies to keep in touch with their children than Sri Lankan migrant workers, which could be attributed to the fact that the use of high technology in communications in Sri Lanka is still not as widespread as in the Philippines. All the same, such means of communication are not available throughout the Philippines due to social inequalities and disparities in income between rural and urban areas (Hugo and Ukwatta, 2010). Another obstacle to keeping communication with their children is the conditions of employment of migrant mothers. Domestic workers, for example, call their children less frequently than other migrant women in other professions, as some employers limit their access to mobile phones (Graham et al., 2012). Furthermore, in their 2021 study, Cardoso et al. uncovered that in Mexico substitute caregivers act as gatekeepers and sometimes do not allow LBCs to contact their mother. This was not the case in the Philippines and Indonesia where children had their own mobile phones and they expressed agency over contacting their mothers (Graham et al., 2012). The study emphasizes that technology places more pressure on migrant mothers to stay in frequent contact with their children compared to fathers, due to gender norms. When mothers fail to do so, it intensifies feelings of guilt and children may blame them for abandonment, worsening their mental health.

## 4 Discussion and analysis

This review highlights the significant impact of maternal migration on the mental health of left-behind children, revealing the complex interplay of various factors that shape these outcomes. The findings underscore the importance of considering the child’s age, the stability and quality of caregiving arrangements, and the extent of communication with migrant mothers, particularly through technological means. However, a more critical analysis of these findings reveals several potential biases and limitations in the reviewed studies.

### 4.1 Critical analysis

One notable limitation is the potential for selection bias, as many studies may focus on specific populations or regions, leading to a lack of generalizability across diverse cultural and socio-economic contexts. Additionally, the cross-sectional nature of many studies limits our understanding of the long-term effects of maternal migration on children’s mental health. The reviewed studies often rely on self-reported data, which can introduce biases related to social desirability or recall accuracy.

Discrepancies between studies, such as differing outcomes for children in similar caregiving situations, may stem from variations in cultural norms, socioeconomic conditions, or methodological differences. For instance, while some studies suggest that communal child-fostering can mitigate the negative effects of maternal migration, others find that it may not fully compensate for the absence of a mother. These discrepancies raise questions about the uniformity of these findings and highlight the need for more standardized research methodologies and longitudinal designs to capture the dynamic nature of these outcomes over time.

The potential implications of these biases and limitations are significant. They suggest that the current understanding of maternal migration’s impact on children may be overly simplified, failing to capture the full complexity of the issue. This underscores the importance of caution when generalizing findings and applying them to policy or practice.

### 4.2 Theoretical implications

The findings of this review also have important theoretical implications, particularly for attachment theory and migration studies. Attachment theory, which traditionally emphasizes the centrality of the mother–child bond, may need to be re-evaluated in the context of maternal migration. The evidence suggesting that children can form secure attachments with multiple caregivers challenges the traditional view of a singular attachment figure and suggests that attachment theory may need to be adapted to account for cultural variations in caregiving practices.

Moreover, these findings contribute to migration studies by highlighting the gendered dimensions of migration and the specific challenges faced by migrant mothers and their children. The feminization of migration complicates traditional gender roles and raises new questions about how these shifts impact family dynamics and child development. The review suggests that migration studies should incorporate more nuanced, intersectional approaches that consider how gender, culture, and socioeconomic status intersect to shape the experiences of migrant families.

### 4.3 Cultural context

The cultural context plays a crucial role in shaping the outcomes of maternal migration, yet this aspect remains underexplored in much of the literature. Different cultural practices around caregiving, such as communal child-rearing or reliance on extended family networks, can significantly influence how children experience maternal absence. For instance, in many African and Southeast Asian societies, communal child-fostering is a common practice, where children are often cared for by extended family members or community members in the absence of their biological parents. In these contexts, children may be more accustomed to forming attachments with multiple caregivers, which can act as a buffer against the negative effects of maternal migration. For example, studies in countries like Ghana and the Philippines have shown that children raised in such communal settings may maintain emotional stability and a sense of security, despite the physical absence of their mothers.

However, this cultural resilience has its limits. The review reveals that even in contexts where communal caregiving is prevalent, challenges persist. The instability that often accompanies cycles of caregiving arrangements—where children may be passed between different relatives or caregivers—can disrupt the development of secure attachments. Secure attachments are crucial for children’s overall well-being, providing them with a stable emotional base from which they can explore the world and develop healthy relationships. Inconsistent caregiving, on the other hand, can lead to feelings of insecurity, abandonment, and emotional distress, which may have long-term psychological impacts. This suggests that while cultural practices may mitigate some of the adverse effects of maternal migration, they cannot entirely eliminate the risks associated with maternal absence.

Expanding the cultural analysis further, it becomes evident that the impact of maternal migration cannot be understood in isolation from broader cultural, economic, and social contexts. For instance, the socioeconomic environment of the destination country plays a critical role in shaping the experiences of migrant mothers. In countries where immigrant labor is devalued and poorly protected by labor laws, migrant mothers may face harsh working conditions, limited access to social services, and prolonged separation from their families due to restrictive immigration policies. These stressors not only affect the well-being of the migrant mothers but also have ripple effects on their children back home, who may experience heightened anxiety and insecurity due to the uncertain and precarious situation of their mothers abroad.

Additionally, cultural attitudes toward immigrants in the destination country can further exacerbate the stress experienced by migrant mothers. In societies where immigrants are viewed with suspicion or hostility, migrant mothers may face social isolation, discrimination, and a lack of support networks, which can compound the emotional strain of being separated from their children. This, in turn, can affect the quality and frequency of communication between the mothers and their children, further weakening the attachment bonds and contributing to negative psychological outcomes for the children.

Attachment theory, which traditionally emphasizes the importance of a consistent and secure attachment figure for a child’s emotional and psychological development, provides a useful framework for understanding the impacts of maternal migration. According to attachment theory, the disruption of the mother–child bond—particularly during critical stages of a child’s development—can lead to attachment insecurity, which is associated with a range of adverse mental health outcomes, including anxiety, depression, and difficulties in forming and maintaining relationships later in life. However, the cultural context challenges some of the assumptions of attachment theory, particularly the idea that the mother is the sole or primary attachment figure. In many non-Western societies, where caregiving is more communal and distributed among several individuals, children may develop multiple attachments that provide them with a sense of security, even in the absence of their mothers. This suggests that attachment theory may need to be adapted or expanded to better account for the diversity of caregiving practices across different cultures.

While cultural practices such as communal child-rearing can offer some protection against the negative effects of maternal migration, they are not a panacea. The impact of maternal migration is deeply intertwined with broader cultural, economic, and social factors, and must be understood within the context of both the home and destination countries. To fully grasp the complexities of these dynamics, it is essential to integrate cultural analysis with attachment theory, recognizing the limitations of applying a one-size-fits-all approach to understanding the psychological impacts of maternal migration on left-behind children. Future research should continue to explore these intersections, providing a more nuanced and culturally informed understanding of how maternal migration affects children across different global landscapes.

## 5 Conclusion

In conclusion, this review reveals the intricate and multifaceted effects of maternal migration on children’s mental health, emphasizing the importance of considering diverse cultural contexts and caregiving practices. Key findings challenge traditional attachment theories, suggesting that the mother–child bond may be more adaptable than previously understood, particularly in non-Western contexts where communal caregiving is prevalent. However, the review also identifies significant gaps in the current literature, particularly the lack of longitudinal studies that explore the long-term psychological effects of maternal migration on children.

For future research, it is crucial to conduct longitudinal studies that track the mental health outcomes of left-behind children over time. Additionally, research should focus on understanding how various cultural practices, socioeconomic conditions, and caregiving arrangements influence these outcomes. Investigating the role of technological communication in maintaining attachment bonds and assessing the differential impacts of maternal versus paternal migration are also important areas for further exploration.

The policy implications of these findings are significant. Governments and organizations should prioritize the mental health and well-being of left-behind children by ensuring access to culturally sensitive mental health services and providing support for stable and nurturing caregiving arrangements. Policies that facilitate family reunification and promote trauma-informed care practices can help mitigate the negative impacts of maternal migration. By addressing these needs, policymakers can help foster resilience and support the overall well-being of children in migrant households, ensuring that the benefits of migration do not come at the expense of children’s mental health.
