# Supplementary material for: Psychological impacts of maternal migration on left-behind children: a cross-cultural review
Source: Front Psychol. 2025 Jan 15;15:1407733. doi: 10.3389/fpsyg.2024.1407733 (PMC11776297; doi:10.3389/fpsyg.2024.1407733)
Supplement: Supplementary file 1 [file Supplementary_file_1.docx]

Appendix 1: Search Strategy

The following outlines the search strategy that was employed to identify relevant articles for this mini-review :

1- Identify Key Words and Phrases:

a- Transitional Families

b- Migrant mothers

c- Maternal Migration

d- Left Behind children

e- Families in transition

f- Children of migrant mothers

g- Attachment

h- Attachment Theory

i- Psychological impact

j- Attachment disorder

k- Migrant workers

l- Mental Health

m- Coping strategies

n- Trauma

o- Cultural practices

2- Select Database:

a. Google Scholar: Google Scholar proved to be an invaluable resource in identifying a significant number of relevant articles for our research. It served as an effective starting point for uncovering pertinent literature on the topic. Additionally, we utilized references cited within the articles we reviewed to expand our search.

b. PsycINFO: While PsycINFO is a well-established database, we found Google Scholar to be more beneficial for our specific topic. The number of articles available in PsycINFO was limited, which influenced our decision to rely more heavily on Google Scholar. Given that our topic is not extensively researched, Google Scholar offered an excellent foundation for our inquiry. Furthermore, we employed the snowball sampling method, utilizing references from the articles we initially identified to uncover additional relevant literature.

3- Construct Search Strings:

a) Use Boolean operators (AND, OR, NOT) to combine keywords, such as:

Left behind children AND Migrant mothers

Migrant Mothers Not Migrant Fathers

Migrant Mothers Not Migrant Parents

b) Wild cards were used, such as:

transit* AND famil*

transit* fami*

transit* family

c) We sometimes combined Boolean logic with wild Cards such like in the following example:

transit* AND (famili* OR "household")

4- Apply Filter

Date Range: 1995 to Present

Language: English

Study Type: Peer-reviewed

5- Conduct the Search in Google Scholar and PsycINFO

6- Screen Results

Inclusion Criteria: Articles focused on international migration, articles of empirical nature, research articles conducted in different countries and different cultural contexts to allow cross country comparison, we included articles with focus on children who have mothers as migrants, children who have fathers are migrants, children who have both parents as migrants, and children who live with both parents to allow accurate comparison.

Exclusion Criteria: Articles that focused on internal migration such as in China, review articles were excluded.

The rationale behind excluding studies that focused on internal migration is that migrants who migrate internally have the chance to conduct regular visits to their children, unlike international migrants who only visit there every two years depending on the contracts they have. This difference may yield different outcomes for children. Furthermore, we excluded review articles and focused on empirical studies, because empirical studies benefit the review in so many ways, among which is making it more reliable and valid because data collected through empirical research is less biased, and it is difficult to verify the accuracy of data in non-empirical research. Also, review articles were excluded as we must revisit the main articles included in the review, as accurately assessing their quality is impossible without thorough review.

7- Review and Select Articles

Review titles and abstracts for relevance.

Retrieve full texts of potentially relevant articles.

Assess full texts against inclusion/exclusion criteria.

8- Data Extraction

Extract data on study design, sample size, mental health outcomes, and key findings.
